# Supplementary material for: Imaging translational control by Argonaute with single-molecule resolution in live cells
Source: Nat Commun. 2022 Jun 10;13:3345. doi: 10.1038/s41467-022-30976-3 (PMC9187665; doi:10.1038/s41467-022-30976-3)
Supplement: Supplementary file 1 — Supplementary Information [file 41467_2022_30976_MOESM1_ESM.pdf]

Imaging translational control by Argonaute with single-molecule resolution in live cells

Supplemental Information:

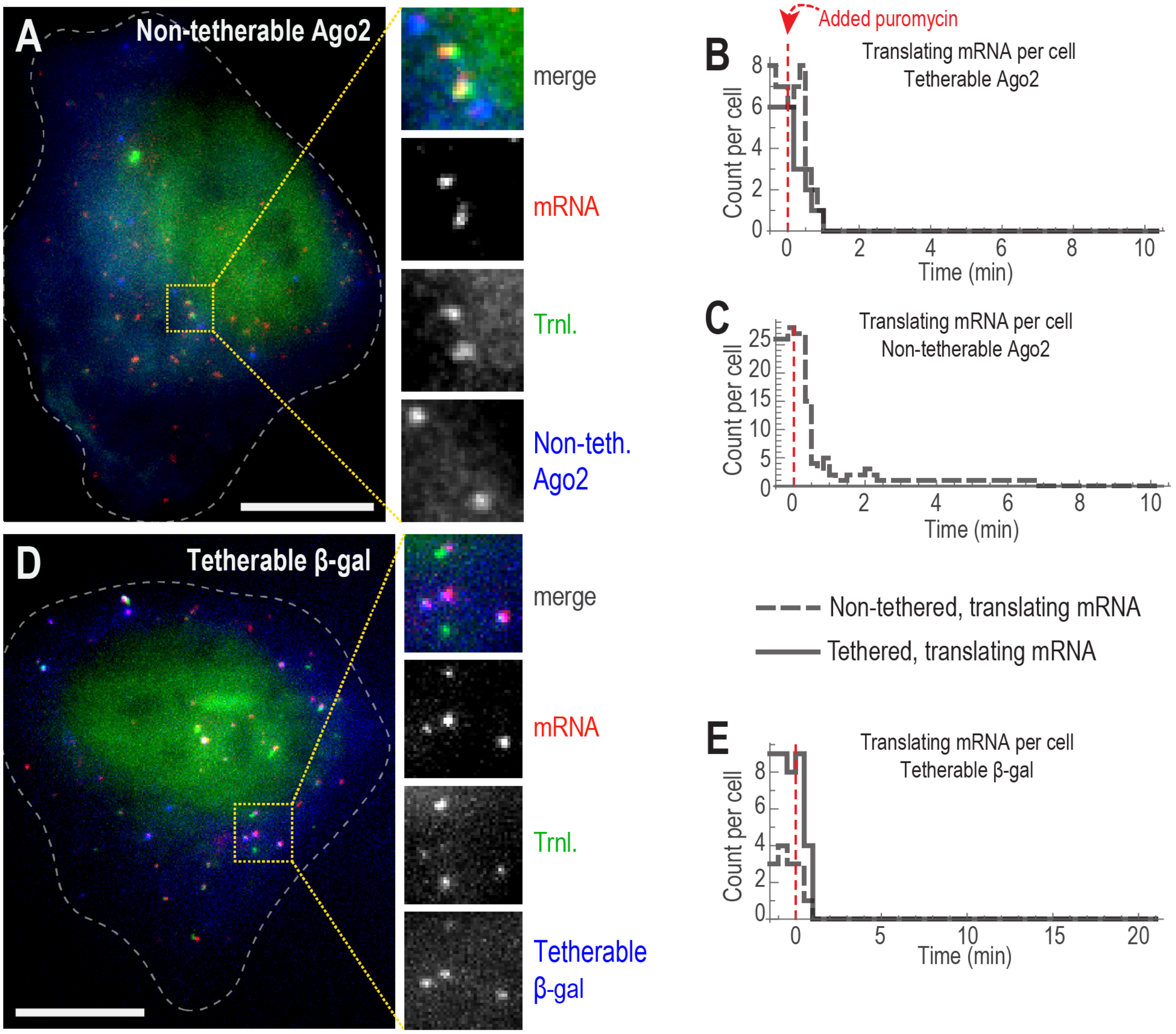

Supplemental Figure 1. The TnT biosensor can be used to simultaneously track translation and tethering to reporter mRNA.

A A representative cell expressing non-tetherable Ago2 (EGFP-Ago2). The image was acquired approximately 4 hours after loading the TnT components (smFLAG-KDM5B-15xBoxB-24xMS2 mRNA reporter, Cy3-FLAG-Fab, and JF646 HaloTag MCP). The dashed line marks the cell boundary. N= 25 mRNA in 1 cell. Scale bars, 10 μm.

B-C Line plots showing the number of mRNA actively being translated in a single, TnT-loaded cell expressing tetherable Ago2 (B) or non-tetherable Ago2 (C), prepared as described above. Cells were treated with puromycin at Time = 0, as indicated by the dashed, vertical red line, and imaged

for 10-20 minutes (min). Solid and dashed lines correspond to tethered and untethered mRNA, respectively.
D A representative cell expressing tetherable β-gal (λN-EGFP-β-gal) and the TnT components. The image was acquired approximately 4 hours after loading the TnT components. The dashed line marks the cell boundary. N= 12 mRNA in 1 cell. Scale bar, 10 μm.
E Same as B,C, but now for a single cell expressing tetherable β-gal.

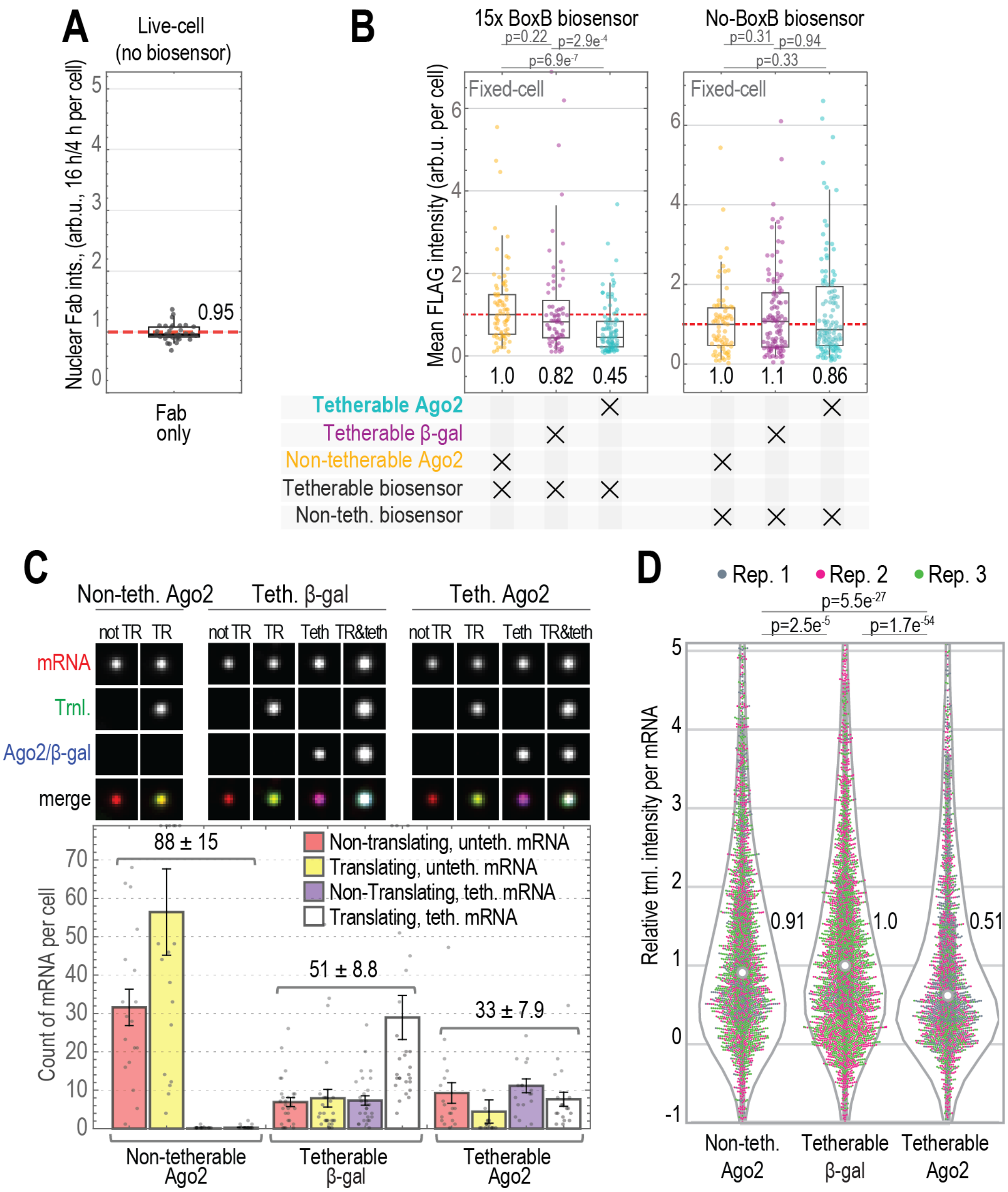

Supplemental Figure 2. Impact of Ago2 tethering on translation.

A Box plot displaying nuclear accumulation of Fab in the absence of the TnT biosensor. Each data point is the ratio of Fab nuclear intensity at 16 hours to 4 hours after Fab loading. N= 35 cells. The boxes (whiskers) show the 25-75% (5-95%) range.

B Box plot showing the mean intensity of the TnT reporter protein KDM5B (as marked by Fab) in fixed cells expressing the TnT biosensor (with or without the BoxB tethering cassette) as well as non-tetherable Ago2, tetherable β-gal, or tetherable Ago2. Cells were fixed 24 hours after TnT biosensor loading and stained with α-FLAG Fab. The mean cellular intensity was measured. N=77 non-tetherable, 67 β-gal-tetherable, and 97 Ago2-tetherable cells (left), and N=70 non-tetherable, 102 β-gal-tetherable, and 114 Ago2-tetherable cells (right). Box plot description same as A. P values were calculated from Bonferroni-corrected Mann-Whitney tests, two sided.

C Bar chart of all mRNA per live, TnT-loaded cells from Fig. 2E (1 of 3 replicates). The mRNA were categorized based on the presence or absence of detectable translation (TR) and/or Ago2/β-gal tethering (Teth) signals. Above, representative background-subtracted and auto-adjusted mean crops from each category (18 x 18 pixels^2; 130 nm/pixel). Note, because very few non-tetherable Ago2 cells had tethering signals, crops showing tethering in this case are not shown. Below, mean counts per cell for each category (color-coded bars). The total number of mRNA particles per cell were N_{total}= 88 ± 15 (mean±SEM), 51 ± 8.8, and 33 ± 7.9 for non-tetherable Ago2, tetherable β-gal and tetherable Ago2, respectively. Error bars show SEM. N= 18 (1513), 28 (1158), 18 (521) cells (mRNA) for non-tetherable Ago2, tetherable β-gal, and tetherable Ago2, respectively.
D Violin plot showing all experimental replicates (discernable by marker color) from the experiment in Fig. 2E. P values were calculated using the Bonferroni-corrected Mann-Whitney test. N = 55 (3,153), 39 (4,947) and 52 (3,959) cells (mRNA) for non-tetherable Ago2, tetherable β-gal, and tetherable Ago2, respectively. P values were calculated from Bonferroni-corrected Mann-Whitney tests, two sided.

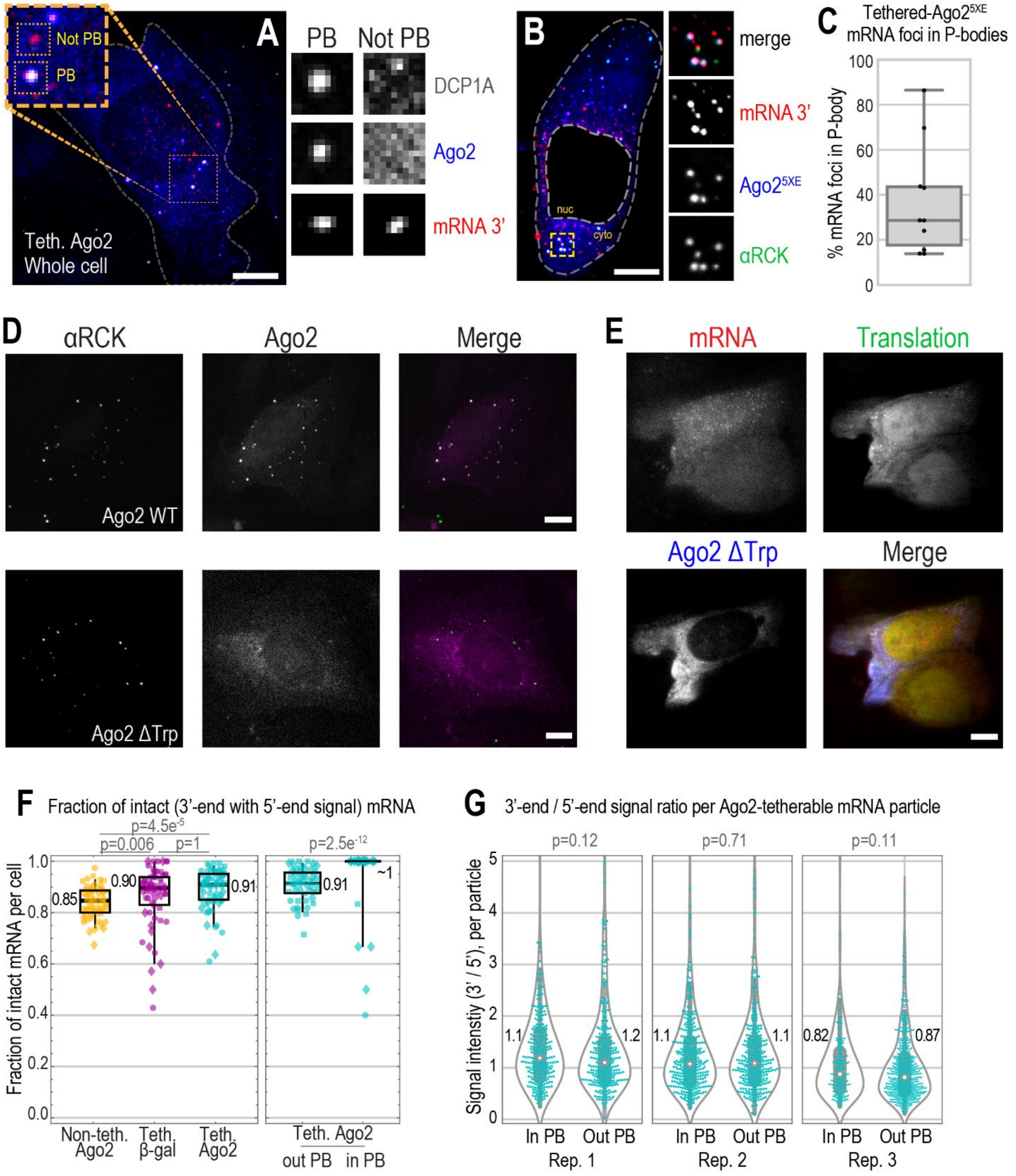

Supplemental Figure 3. TnT biosensors in cells have 5' and 3' smiFISH signals and Ago2^{5XE}-tethered TnT biosensors go to P-bodies.

A Fixed cell expressing tetherable Ago2 and reporter mRNA (smFLAG-KDM5B-15xBoxB-24xMS2) depicting colocalization of Ago2-tethered mRNA to P-bodies. The 3' reporter mRNA ends were labeled with smiFISH probes, and P-bodies were stained with α-DCP1A antibodies. N= 10 cells. Scale bar, 10 μm.

B Fixed cell expressing tetherable Ago2^{5XE} and reporter mRNA (smFLAG-KDM5B-15xBoxB-24xMS2) depicting colocalization of Ago2^{5XE}-tethered mRNA to P-bodies. The 3’ reporter mRNA ends were labeled with smiFISH probes, and P-bodies were stained with α-RCK antibodies. N= 10 cells. Scale bar, 10 μm.
C Box plot of percentage of mRNA localized to P-bodies. Reporter mRNA 3’UTR was stained using smiFISH probes and P-bodies were stained with α-RCK antibodies. N= 5 cells. The boxes (whiskers) show the 25-75/% (full) range.
D Fixed cell expressing tetherable Ago2 WT (Top) and tetherable Ago2 ΔTrp (bottom) from two replicates. P-bodies were stained with α-RCK antibodies. Scale bar, 10 μm.
E Live cell expressing tetherable Ago2 ΔTrp and reporter mRNA (smFLAG-KDM5B-15xBoxB-24xMS2) from two replicates acquired 8 hours after loading the TnT components (mRNA reporter, Cy3-FLAG-Fab, and JF646 HaloTag MCP). Scale bar, 10 μm.
F Box plots quantifying fractions of mRNA per cell detected by 3’ smiFISH and 5’ smiFISH signals (yellow, non-tetherable Ago2; purple, tetherable β-gal; cyan, tetherable Ago2; shapes mark 1 of 3 replicates). Left, tetherable Ago2 versus controls. N= 56 non-tetherable Ago2, 61 tetherable β-gal, and 63 tetherable Ago2 cells. Right, tetherable Ago2 signals in P-bodies (PBs) versus out. N= 50 tetherable Ago2 Cells. P values were calculated from Mann-Whitney tests (left was Bonferroni-corrected). The boxes (whiskers) show the 25-75% (5-95%) range.
G Violin plots showing the ratio of 3’ to 5’ smiFISH signals per mRNA focus (including multi-mRNA clusters) in or out of PBs in all cells expressing tetherable Ago2 over three replicates. The median ratio value per replicate was calculated. N= 25 (3224), 24 (1839), 22 (2166) cells (mRNA) for non-tetherable Ago2, tetherable β-gal, and tetherable Ago2 respectively. P values were calculated from Mann-Whitney tests.

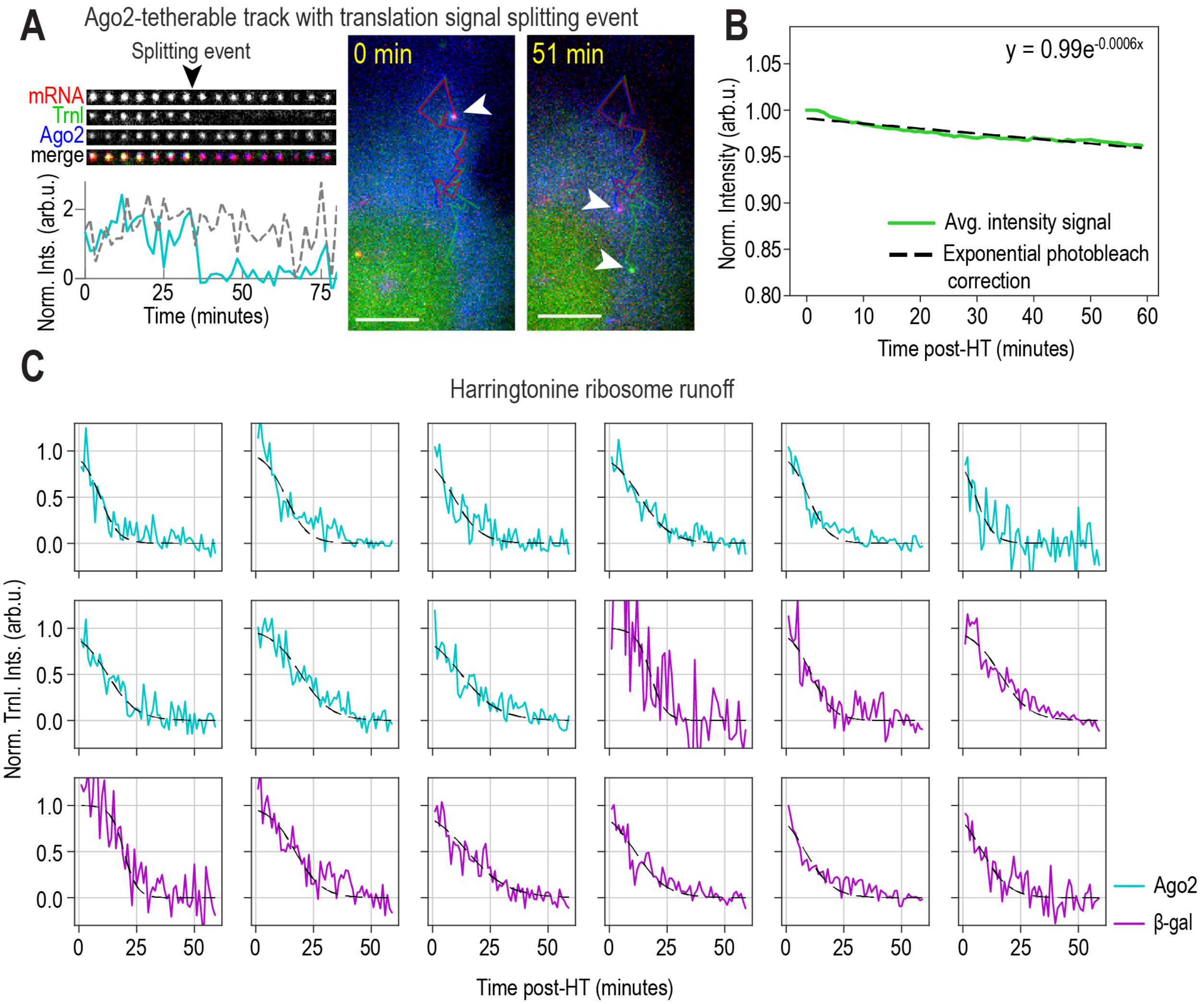

Supplemental Figure 4. Example event showing the splitting of mRNA and translation signals and Harringtonine experiments analyses.

A One of two example tracks from Fig. 4C where the translation signal split from the mRNA. Plotted as in Fig. 4E. Right, two sample images are shown with the tracks overlaid (mRNA, red; translation, green; Ago2, blue). Arrows indicate the current location of the biosensor at the displayed times (yellow text). N= 2 tracks. Scale bar, 5 µm.

B Signal photobleaching of translation signals over time in Harringtonine (HT) ribosome runoff assays for both Ago2 and β-gal was measured, averaged and normalized. The data was fit to a single exponential decay to correct for photobleaching in the experiments.

C Translation signals from single HT ribosome runoff assays for Ago2 and β-gal were normalized, plotted, and fit to a phenomenological model (Eq. 3 in the Materials and Methods) to determine the average ribosome runoff rates plotted in Fig. 4I (Ago2, cyan; β-gal, magenta; fit, dashed black line).

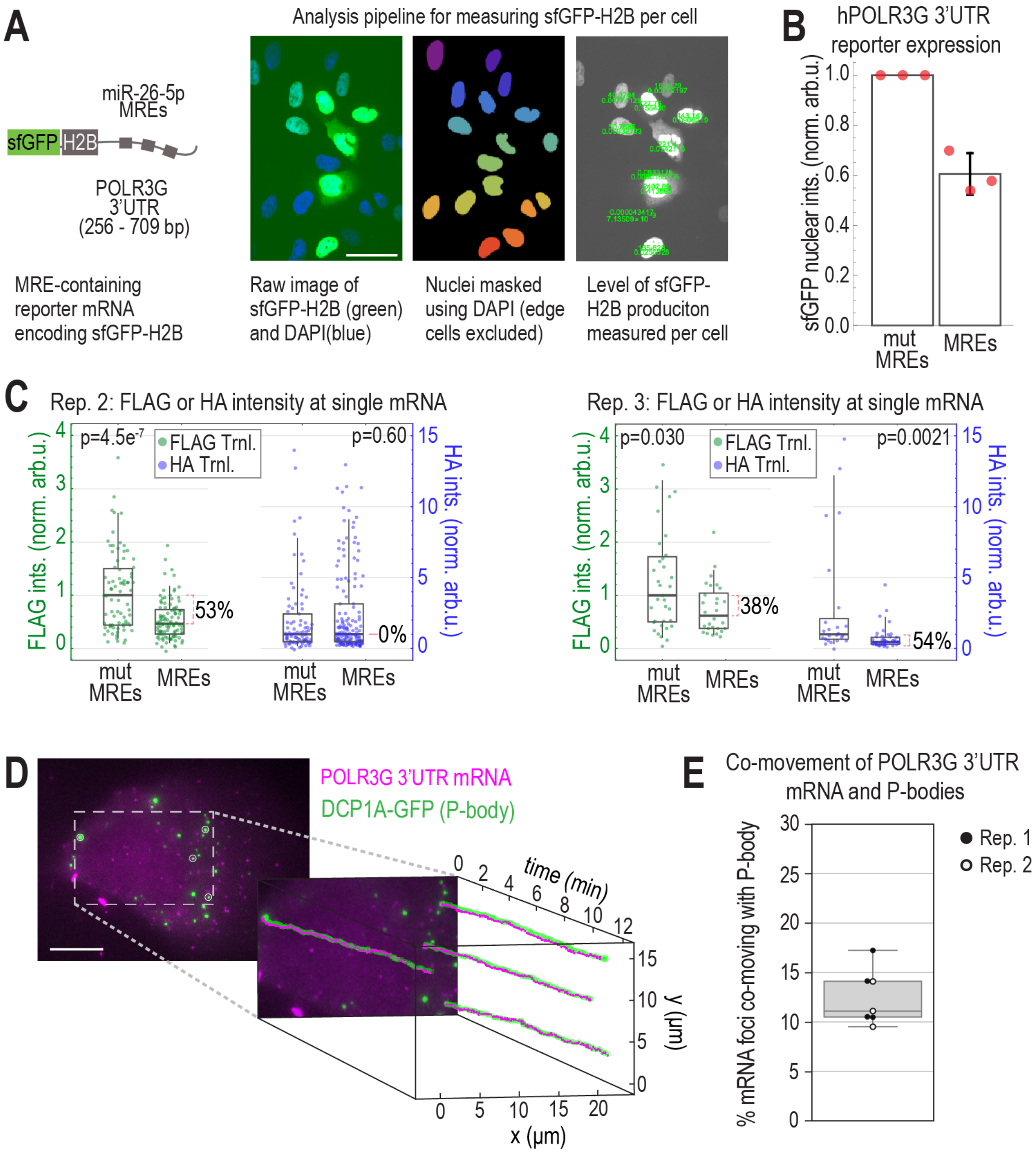

Supplemental Figure 5. miRNA-directed Ago targeting represses translation at the whole-cell and single-mRNA levels.

A Schematic of the MRE-containing sfGFP-H2B reporter and analysis pipeline. Scale bar, 25 µm.
B The nuclear GFP intensity of cells expressing sfGFP-H2B-hPOLR3G 3'UTR with endogenous or mutated (mut) miRNA Response Elements (MREs) was quantified. Points represent the medians per replicate experiment. N = 366, 325, and 1258 nuclei for each replicate experiment for the mutated-MRE-containing reporter (mut MREs) and N = 883, 458, and 1803 nuclei for each replicate experiment for the MRE-containing reporter (MREs). Error bar represents a 95% confidence interval.
C Box plots showing two additional replicate experiments like the one shown in Fig. 5C. Left, N = 79 mut and N = 99 MRE green spots for FLAG; N = 85 mut and N = 153 MRE blue spots for HA. Right, N = 35 mut and N = 28 MRE green spots for FLAG; N = 24 mut and N = 44 MRE blue spots for HA.

The boxes (whiskers) show the 25-75% (5-95%) range. P values were calculated using Mann-whitney tests, two sided.

D Representative live cell expressing the smFLAG-KDM5B-hPOLR3G 3'UTR mRNA reporter (purple) and DCP1A-eGFP (green). Co-movement of 4 mRNAs were tracked for 12 minutes and the positions of the mRNA and P-bodies (identified through DCP1A) were plotted over time at intervals of 6 seconds. N=5 cells in 2 repetitions. Scale bar, 10 μm.

E Box plot of the percentage of smFLAG-KDM5B-hPOLR3G 3'UTR mRNA foci co-moving with P-bodies for at least 30 seconds. N= 5 cells. The boxes (whiskers) show the 25-75% (full) range.
