# Supplementary material for: Imaging translational control by Argonaute with single-molecule resolution in live cells
Source: Nat Commun. 2022 Jun 10;13:3345. doi: 10.1038/s41467-022-30976-3 (PMC9187665; doi:10.1038/s41467-022-30976-3)
Supplement: Supplementary file 3 — Description of Additional Supplementary Files [file 41467_2022_30976_MOESM3_ESM.pdf]

Description of Additional Supplementary Files

File Name: Supplementary Movie 1
Description: Simultaneous imaging of single-molecule translation and tethering with the TnT biosensor. A video showing a representative cell loaded with the TnT components (plasmids encoding λN-EGFP-Ago2, smFLAG-KDM5B-15xBoxB-24xMS2 mRNA reporter, Cy3-FLAG-Fab, and JF646 HaloTag MCP). The video was acquired at 0.5 frames/second for 40 seconds total (blue, tetherable Ago2; green, translation; red, mRNA). The circles mark single mRNA being (i) translated and Ago2-tethered, (ii) translated and Ago2-untethered, or (iii) non-translated and Ago2-tethered. Scale bar, 10 µm.

File Name: Supplementary Movie 2
Description: TnT biosensor translation signals disappear upon puromycin treatment. A video showing a representative cell loaded with the TnT components (plasmids encoding λN-EGFP-Ago2, smFLAG-KDM5B-15xBoxB-24xMS2 mRNA reporter, Cy3-FLAG-Fab, and JF646-stained MCP) and treated with puromycin. The video was acquired at 0.1 frames/second for 30.67 minutes total (blue, tetherable Ago2; green, translation; red, mRNA). 50 mg/ml puromycin was added at Time = 0 (frame 5). Scale bar, 10 µm.

File Name: Supplementary Movie 3
Description: Translationally-silenced TnT biosensors cluster and coalesce with other mRNA foci over hours. A video showing a translationally-silenced, Ago2-tethered TnT biosensor clustering and coalescing with other mRNA foci over hours. The video comes from a representative cell 4 - 16.5 hours after loading the TnT components (plasmids encoding λN-EGFP-Ago2 and smFLAG-KDM5B-15xBoxB-24xMS2 mRNA reporter, Cy3-FLAG-Fab, and JF646 HaloTag MCP). The cell was imaged every 30 minutes for 12 hours (blue, tetherable Ago2; green, translation; red, mRNA). Scale bar, 10 µm.

File Name: Supplementary Movie 4
Description: Ago2 tethering silences translation gradually at a single mRNA. A video showing a sample track of a single TnT biosensor in cells after loading TnT components (plasmids encoding λN-EGFP-Ago2, smFLAG-KDM5B-15xBoxB-24xMS2 mRNA reporter, Cy3-FLAG-Fab, and JF646 HaloTag MCP). The video was acquired at 0.1 frames/second in the mRNA channel and 0.01 frames/second in the translation (green) and Ago2 tethering (blue) channels for a total of 75 minutes. The frame size is 0.6 µm x 0.6 µm.

File Name: Supplementary Movie 5
Description: Long-term silencing of Ago2-tethered mRNA. A video showing a sample track of a single TnT biosensor in cells after loading TnT components (plasmids encoding λN-EGFP-Ago2, smFLAG-KDM5B-15xBoxB-24xMS2 mRNA reporter, Cy3-FLAG-Fab, and JF646 HaloTag MCP). The video was acquired at 0.88 frames/second in the mRNA channel and 0.088 frames/second in the translation (green) and Ago2 tethering (blue) channels for a total of 79.2 minutes. The frame size is 0.46 µm x 0.46 µm.

File Name: Supplementary Movie 6
Description: Ago2-tethered mRNA loses translation signal in a rare splitting event at a single mRNA. A video showing a sample track of a single TnT biosensor in cells after loading TnT components (plasmids encoding λN-EGFP-Ago2, smFLAG-KDM5B-15xBoxB-24xMS2 mRNA reporter, Cy3-FLAG-Fab, and JF646 HaloTag MCP). The video was acquired at 0.1 frames/second in the mRNA channel and 0.01 frames/second in the translation (green) and Ago2 tethering (blue) channels for a total of 75 minutes. The frame size is 1.15 µm x 1.15 µm.

File Name: Supplementary Movie 7
Description: Sample Harringtonine runoff experiment in cells expressing tetherable Ago2. A video showing a sample cell after loading TnT components (plasmids encoding λN-EGFP-Ago2, smFLAG-KDM5B-15xBoxB-24xMS2 mRNA reporter, Cy3-FLAG-Fab, and JF646-stained MCP). Harringtonine was added at Time = 0 (frame 5). The video was acquired at 1 frames/minute for a total of 65 minutes (blue, tetherable Ago2; green/grayscale, translation; red, mRNA). Scale bar, 10 µm.

File Name: Supplementary Movie 8
Description: Tracking the translation of single biosensors containing either endogenous and mutated MREs in the same cell. A video of a sample cell expressing modified biosensors with either 10 FLAG epitopes and endogenous MREs or 10 HA epitopes and mutated MREs. The cells were loaded with these plasmids along with Cy3 α-FLAG Fab to label FLAG translation (green), GFP-tagged α-HA Frankenbody to label HA translation (blue), and JF646 HaloTag MCP to label mRNA (red). The video was acquired at 0.5 frames/second for a total of 40. Scale bar, 10 µm.
