# Supplementary material for: Imaging translational control by Argonaute with single-molecule resolution in live cells
Source: Nat Commun. 2022 Jun 10;13:3345. doi: 10.1038/s41467-022-30976-3 (PMC9187665; doi:10.1038/s41467-022-30976-3)
Supplement: Supplementary file 12 — Reporting Summary [file 41467_2022_30976_MOESM12_ESM.pdf]

Corresponding author(s): Timothy Stasevich

Last updated by author(s): May 10, 2022

Reporting Summary

Nature Research wishes to improve the reproducibility of the work that we publish. This form provides structure for consistency and transparency in reporting. For further information on Nature Research policies, see our Editorial Policies and the Editorial Policy Checklist.

Statistics

For all statistical analyses, confirm that the following items are present in the figure legend, table legend, main text, or Methods section.

n/a	Confirmed	
☐	☒	The exact sample size (*n*) for each experimental group/condition, given as a discrete number and unit of measurement
☐	☒	A statement on whether measurements were taken from distinct samples or whether the same sample was measured repeatedly
☐	☒	The statistical test(s) used AND whether they are one- or two-sided<br>*Only common tests should be described solely by name; describe more complex techniques in the Methods section.*
☐	☒	A description of all covariates tested
☐	☒	A description of any assumptions or corrections, such as tests of normality and adjustment for multiple comparisons
☐	☒	A full description of the statistical parameters including central tendency (e.g. means) or other basic estimates (e.g. regression coefficient) AND variation (e.g. standard deviation) or associated estimates of uncertainty (e.g. confidence intervals)
☐	☒	For null hypothesis testing, the test statistic (e.g. *F*, *t*, *r*) with confidence intervals, effect sizes, degrees of freedom and *P* value noted<br>*Give P values as exact values whenever suitable.*
☒	☐	For Bayesian analysis, information on the choice of priors and Markov chain Monte Carlo settings
☒	☐	For hierarchical and complex designs, identification of the appropriate level for tests and full reporting of outcomes
☒	☐	Estimates of effect sizes (e.g. Cohen's *d*, Pearson's *r*), indicating how they were calculated

Our web collection on statistics for biologists contains articles on many of the points above.

Software and code

Policy information about availability of computer code

Data collection	Microscopy images were collected using Micro Manager 1.4.22
Data analysis	ImageJ (v2.0.0) was used to post-process microscopy images. Mathematica (v12.0.0.0), python3 and Napari (v0.4.2), were used to analyze the image data. Mathematica and Seaborn (v0.1.1.0) were used to generate graphs and plots.<br>Custom code available on Github [https://github.com/Colorado-State-University-Stasevich-Lab/single-molecule-tracking-python].

For manuscripts utilizing custom algorithms or software that are central to the research but not yet described in published literature, software must be made available to editors and reviewers. We strongly encourage code deposition in a community repository (e.g. GitHub). See the Nature Research guidelines for submitting code & software for further information.

Data

Policy information about availability of data

All manuscripts must include a data availability statement. This statement should provide the following information, where applicable:

- Accession codes, unique identifiers, or web links for publicly available datasets
- A list of figures that have associated raw data
- A description of any restrictions on data availability

The raw and processed data and images generated in this study have been deposited in the Figshare database under “Datasets associated with ‘Imaging translational control by Argonaute with single-molecule resolution in live cells’” [https://doi.org/10.6084/m9.figshare.c.5395800].

Field-specific reporting

Please select the one below that is the best fit for your research. If you are not sure, read the appropriate sections before making your selection.

☒ Life sciences ☐ Behavioural & social sciences ☐ Ecological, evolutionary & environmental sciences

For a reference copy of the document with all sections, see nature.com/documents/nr-reporting-summary-flat.pdf

Life sciences study design

All studies must disclose on these points even when the disclosure is negative.

Sample size	Sample sizes were constrained by the number of cells we could find, image, and quantify per experiment using our microscope setups. In each live experiment, we could image from 3-30 live cells, from which we could make between ~500 and ~1,500 live-cell single molecule measurements total.<br>In each fixed cell experiment, we could image from 40-1,800 fixed cells, from which we could make between ~3,000 and ~4,000 fixed-cell single molecule measurements total.
Data exclusions	No data were excluded
Replication	Replication was performed by repeating each experiment 2-4 times. Replication was always successful except for 1 replicate shown in Sup. Fig. 5C, which is noted in the text as follows: "Moreover, the overall 37-57% reduction in translation in all but one replicate is in good agreement with the 40-60% reduction we observed when tethering Ago2 to the original TnT biosensor (Fig. 2E)."
Randomization	Randomization is not relevant to our study as the analysis of microscopy data was automated in an unbiased manner. Parameters, such as thresholding, were consistent for mRNA, translation, Ago2, and P-body markers in each experiment.
Blinding	Blinding was performed during data collection and analysis for 2 of 3 of all experimental repeats involving tetherable Ago2 versus tetherable β-gal versus Non-tetherable Ago2. In all other experiments, consistent thresholds for particle detection and subsequent analyses were used to remove biases.

Reporting for specific materials, systems and methods

We require information from authors about some types of materials, experimental systems and methods used in many studies. Here, indicate whether each material, system or method listed is relevant to your study. If you are not sure if a list item applies to your research, read the appropriate section before selecting a response.

Materials & experimental systems

n/a	Involved in the study	
☐	☒	Antibodies
☐	☒	Eukaryotic cell lines
☒	☐	Palaeontology and archaeology
☒	☐	Animals and other organisms
☒	☐	Human research participants
☒	☐	Clinical data
☒	☐	Dual use research of concern

Methods

n/a	Involved in the study	
☒	☐	ChIP-seq
☒	☐	Flow cytometry
☒	☐	MRI-based neuroimaging

Antibodies

Antibodies used	1:500 dilution of α-RCK antibody (MBL, PD009, Lot 033); 1:500 dilution of α-DCP1A antibody (abcam, ab47811, Lot GR3267649-3); 1:2000 dilution of α-Rabbit antibody (Jackson ImmunoResearch, 711-476-152, Lot 143770), and α-FLAG antibody (Wako, 012-22384, Anti DYKDDDDK, mouse IgG2b, Lot SAN4130, clone No. 1E6)
Validation	α-FLAG was validated by the manufacturer 's website: https://labchem-wako.fujifilm.com/us/product/detail/W01W0101-2238.html<br>α-DCP1A was validated by the manufacturer 's website:https://www.abcam.com/dcp1a-antibody-ab47811.html<br>α-RCK was validated by the manufacturer 's website: https://www.mblintl.com/products/PD009/<br>α-Rabbit was validated by the manufacturer's website: https://www.jacksonimmuno.com/catalog/products/711-476-152

Eukaryotic cell lines

Policy information about cell lines

Cell line source(s)	Only U2OS cells were used in this study, and they came from ATCC.

Authentication	The cell lines were verified from ATCC at time of purchase. Cells were authenticated by STR profiling by ATCC and morphology.
Mycoplasma contamination	The cell line used tested negative for mycoplasma.
Commonly misidentified lines (See ICLAC register)	No misidentified cell lines were used in the study.
